# Supplementary material for: Two distinct archaeal type IV pili structures formed by proteins with identical sequence
Source: Nat Commun. 2024 Jun 14;15:5049. doi: 10.1038/s41467-024-45062-z (PMC11178852; doi:10.1038/s41467-024-45062-z)
Supplement: Supplementary file 1 — Supplementary Information [file 41467_2024_45062_MOESM1_ESM.pdf]

Supplementary Information

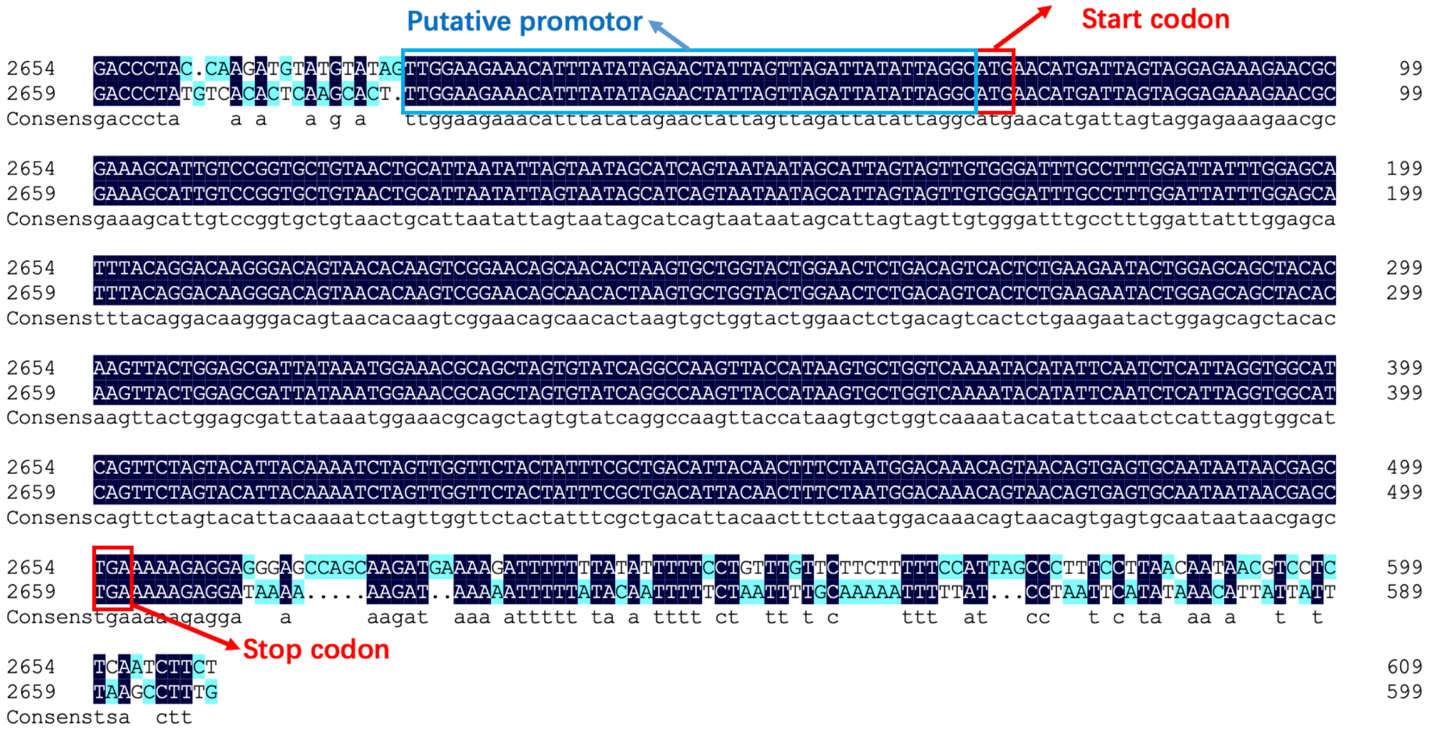

Supplementary Figure 1. Sanger sequencing confirmed that the two pilin genes, SiRe_2654 and SiRe_2659, are identical. The complete sequencing results are presented, with start and stop codons annotated.

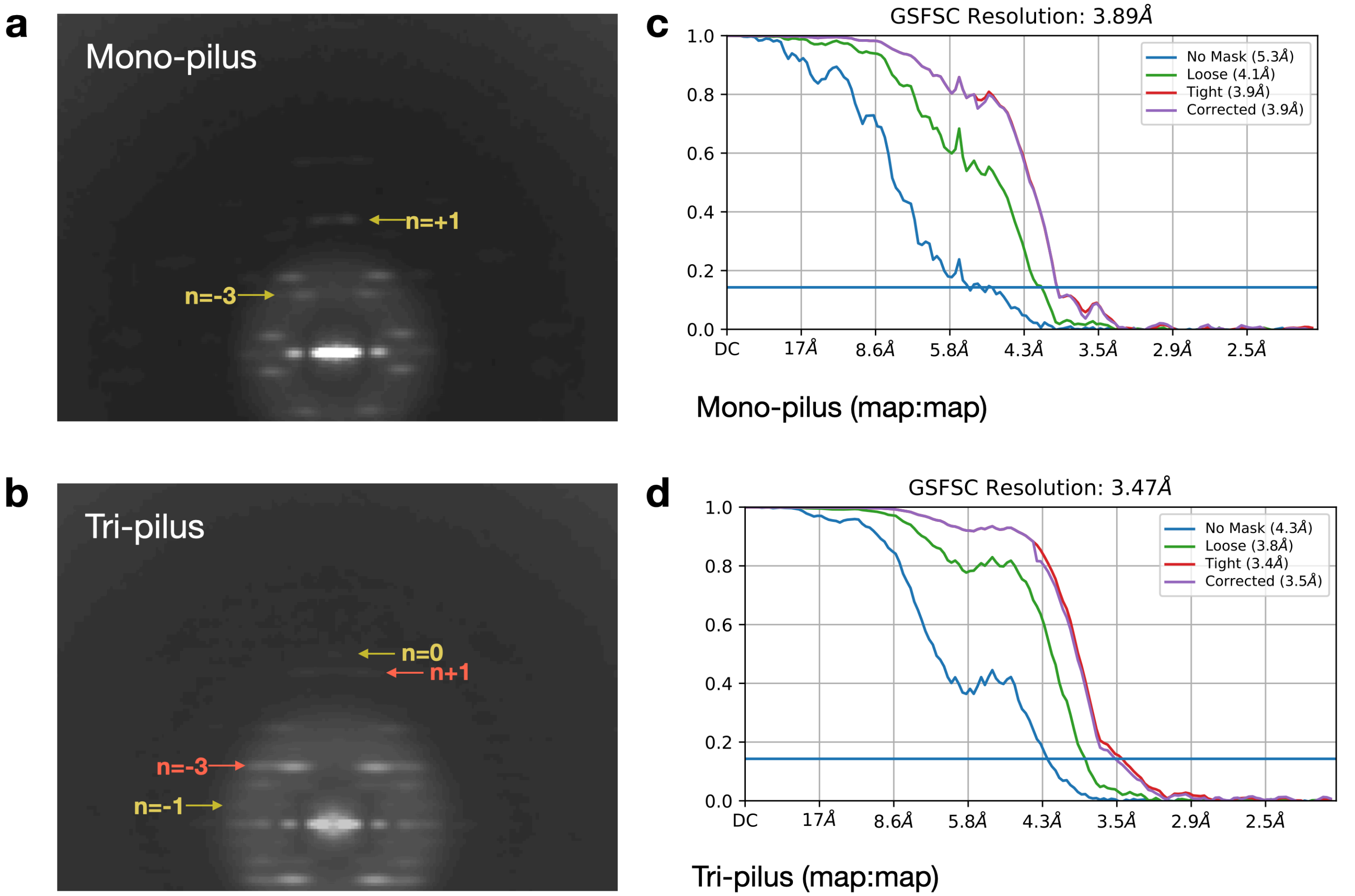

Supplementary Figure 2. Average power spectra and Fourier Shell Correlation (FSC) calculations of mono-pilus and tri-pilus

Average power spectra of particles from 2D class average of mono-pilus (**a**) and tri-pilus (**b**). The Bessel orders of the layer lines used to determine the global helical symmetry are labeled in yellow. For tri-pilus, the layer-lines corresponding to the features generated from the inner helices are labeled in red. The map:map "gold-standard FSC" curves are shown for the mono-pilus (**c**) and tri-pilus (**d**).

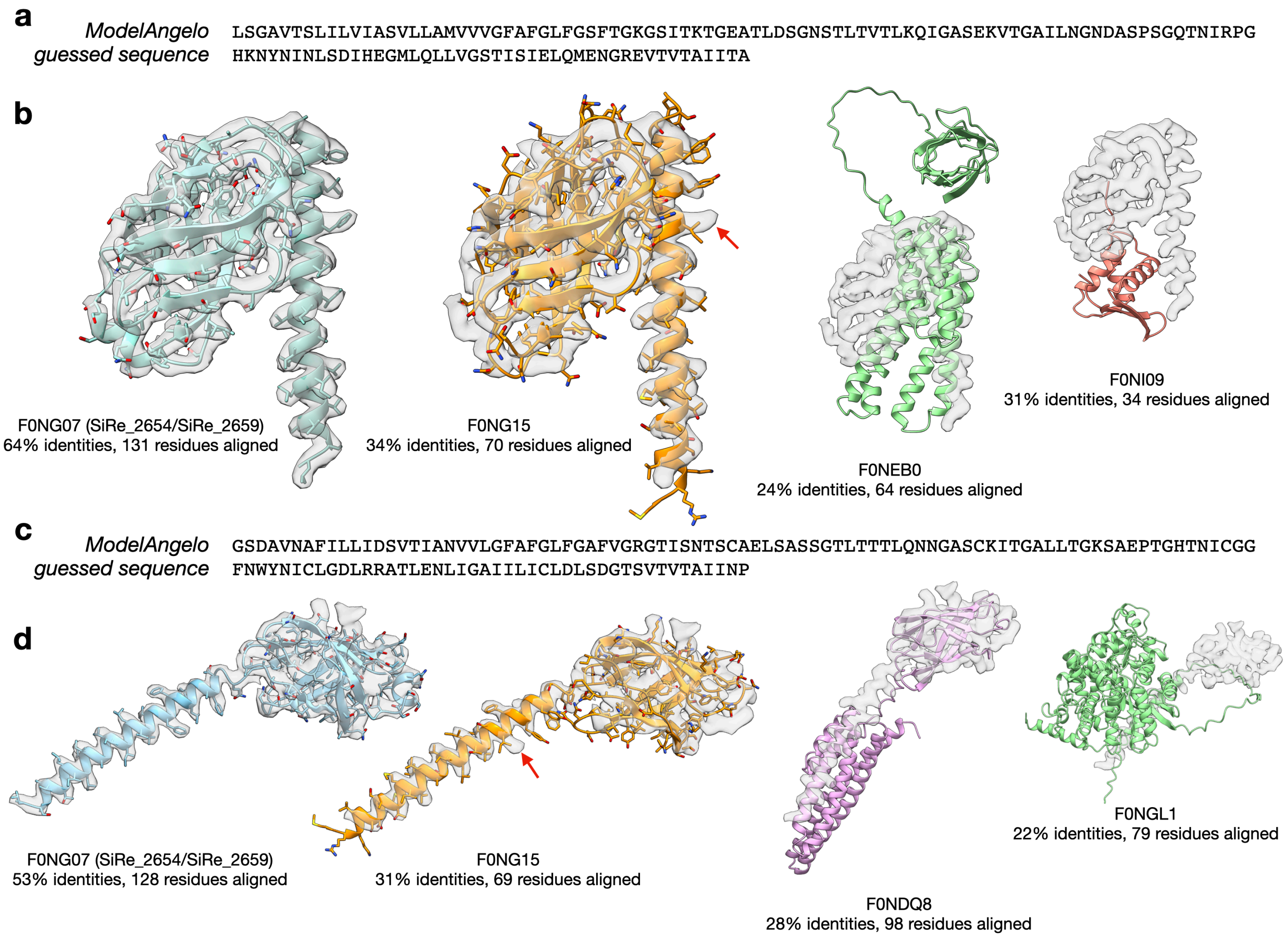

Supplementary Figure 3. Identification of pilin using ModelAngelo and cryo-EM modeling

(**a**) Pilin sequence as predicted by ModelAngelo based on the tri-pilus cryo-EM map. (**b**) Top hits from the BLAST results, derived by searching the predicted sequence against *Sulfolobus islandicus REY15A*, are displayed. Both their sequence identity to the predicted sequence and the number of aligned residues are indicated. A red arrow highlights a notable disagreement between the AlphaFold model and the cryo-EM map. (**c**) Pilin sequence as predicted by ModelAngelo based on the mono-pilus cryo-EM map. (**d**) A similar analysis to (**b**) but uses the sequence predicted using the mono-pilus map.

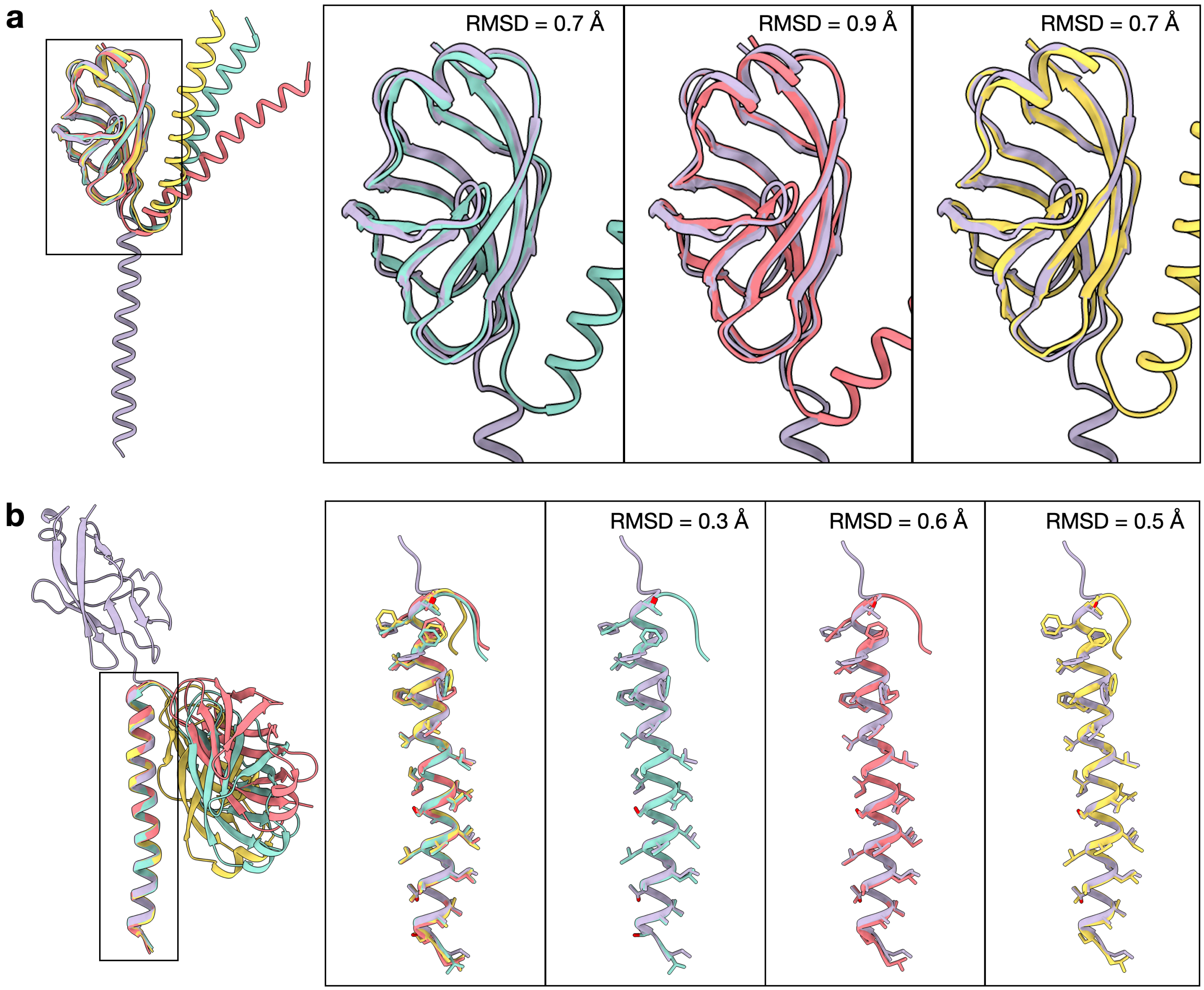

Supplementary Figure 4. Alignment of C-terminal and N-terminal domains of mono-pilin and tri-pilins

(**a**) Alignment of the C-terminal Ig-like globular domains is depicted, comparing the mono-pilin (lavender) with each of the three tri-pilins (A, cyan; B, red; C, yellow). Insets provide a magnified view of region (**a**), focusing on the mono-pilin and a single tri-pilin subunit, along with the labeled backbone RMSD (Root Mean Square Deviation).

(**b**) Alignment of each of the three tri-pilins to the mono-pilin by the N-terminal long helix, following the same visualization approach as in panel (**a**). Sidechains for residues 13-45 are additionally shown.

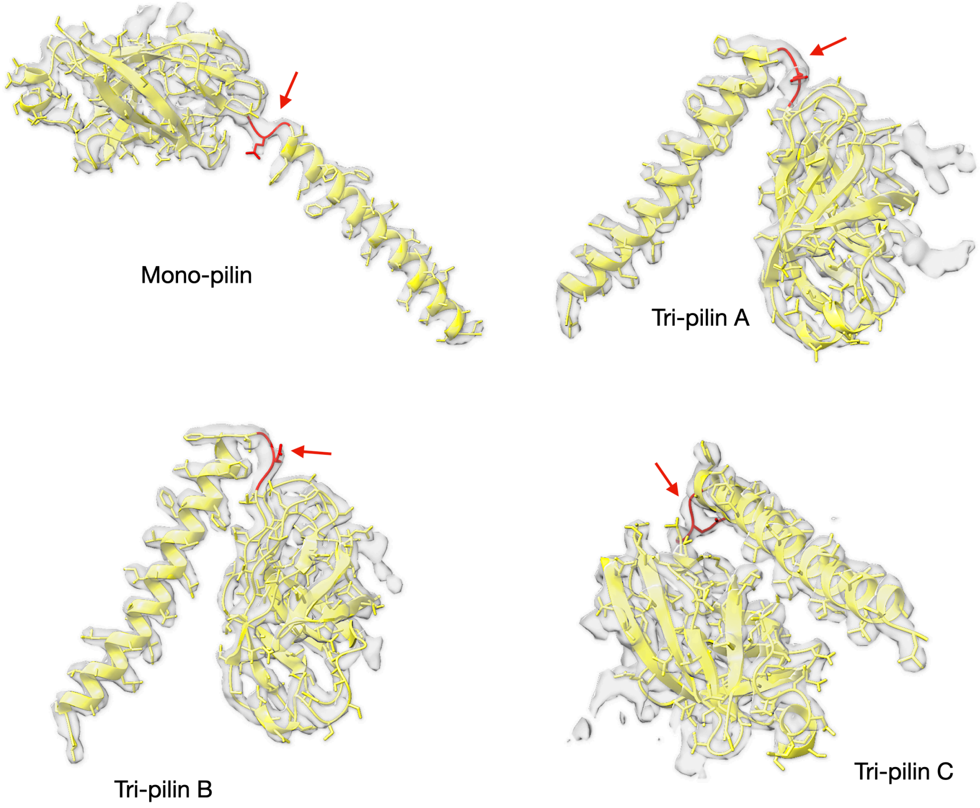

Supplementary Figure 5. Cryo-EM densities of the linker region.

The cryo-EM densities for the pilin subunits are presented. Pilin models are shown in yellow ribbon, with side chains displayed. The linker “Gly-Gln-Gly” regions are highlighted in red.

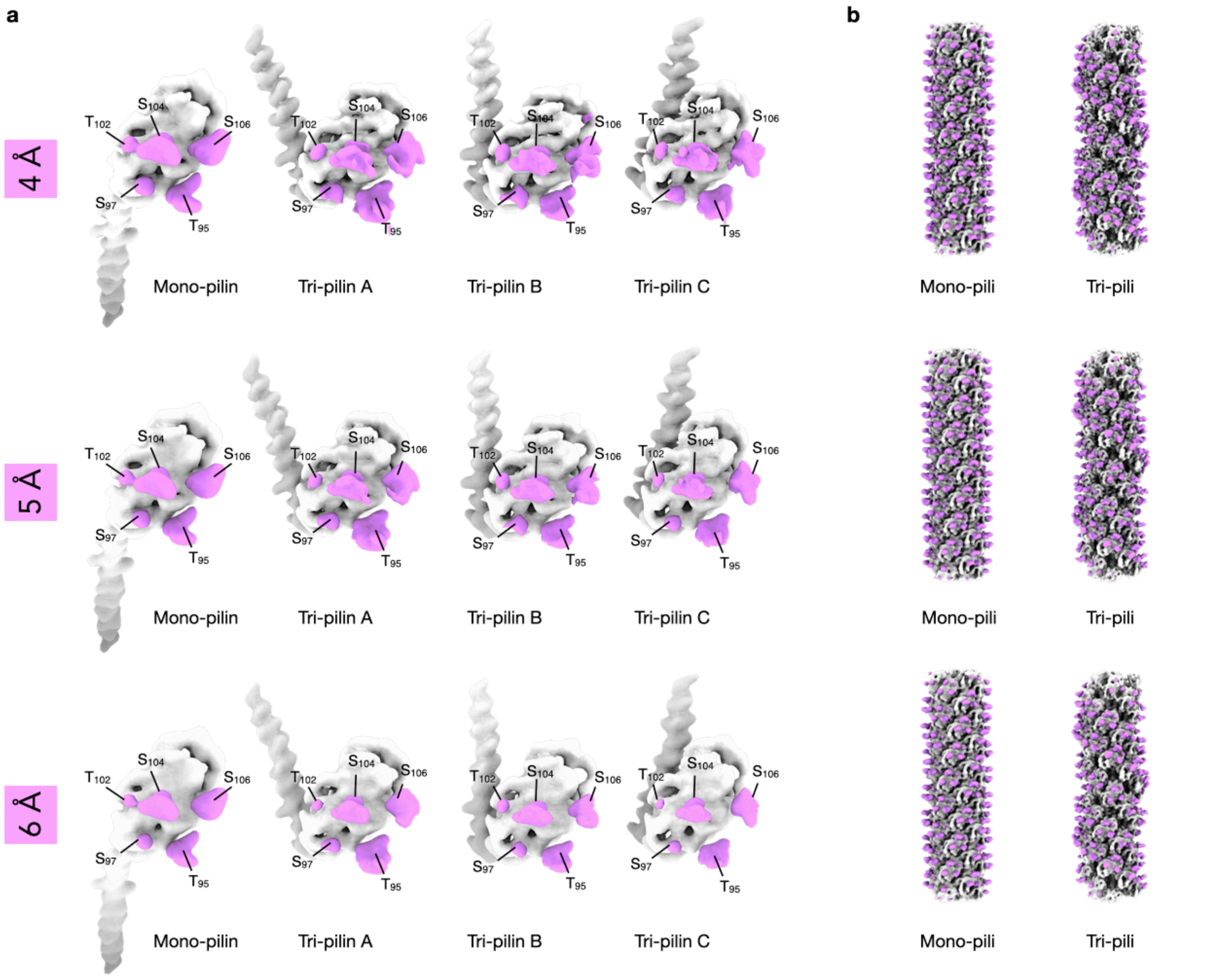

Supplementary Figure 6. Levels of surface glycosylation of mono and tri-pilus, filtered at different resolution.

(**a**) Cryo-EM map densities due to post-translational modifications on pilin subunits. All volumes were filtered to 4, 5, and 6 Å, respectively. The densities accounted for the protein part (grey) were segmented out using a mask generated from the refined atomic model. The extra experimental density after subtracting the protein part, presumably coming from O-linked sugars on Ser or Thr residues, are colored in magenta. The amino acid side chains with extra densities are labeled.

(**b**) Surface glycosylation of the mono-pilus (left) and tri-pilus (right) filtered at the same resolution as in (a).

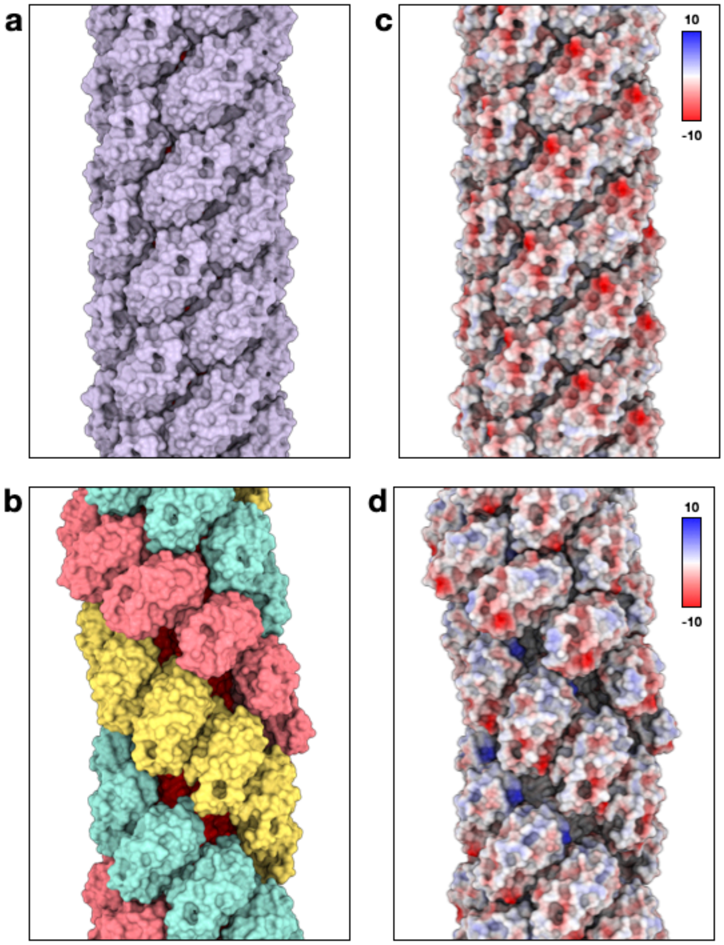

Supplemental Figure 7. Solvent-excluded and electrostatic surfaces of the mono-pilus and tri-pilus

(**a**) Molecular solvent-excluded surface of the mono-pilus. The inner helices are colored in red, but cannot be seen due to their shielding by the outer domains.

(**b**) Molecular solvent-excluded surface of the tri-pilus. The inner helices of all pilins are colored in red. Electrostatic surface of the mono-pilus (**c**) and the tri-pilus (**d**). Scale bar displays the color-coding of the electrostatic potential in units kcal·mol^{-1}·e^{-1} at 298 K.

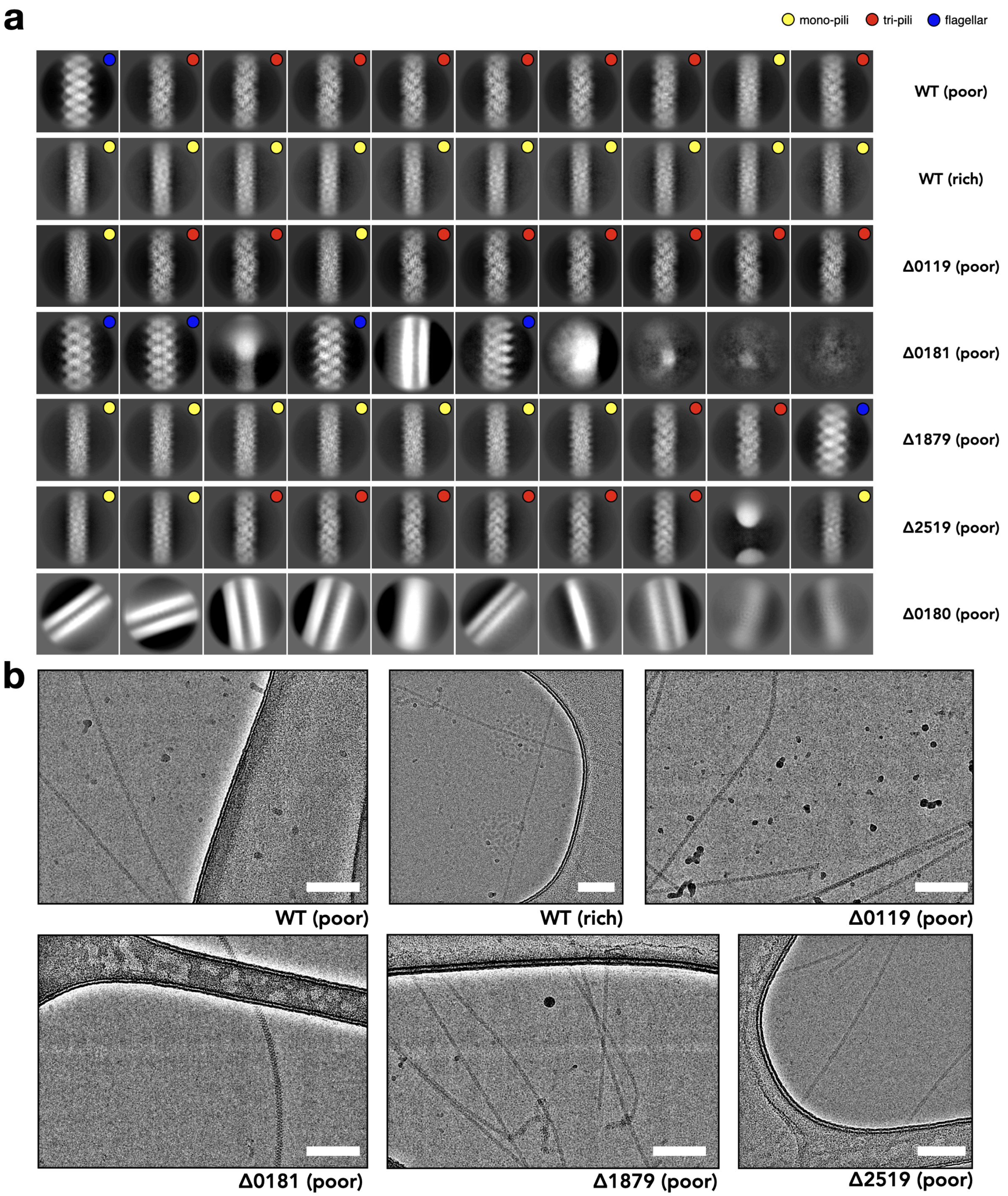

Supplementary Figure 8. Pili production under different media and ATPase knockout strains
(**a**) The top ten 2D classes for datasets collected in Fig 6c. In total there are 50 classes, and after the first 10, there are more classes containing carbon edges etc. Also, each class may have different number of particles. The filament species identified based on the 2D averages, and their corresponding power spectrum are labeled on the upper right.
(**b**) Representative micrographs of the strains illustrated in (**a**). Scale bars, 100 nm.

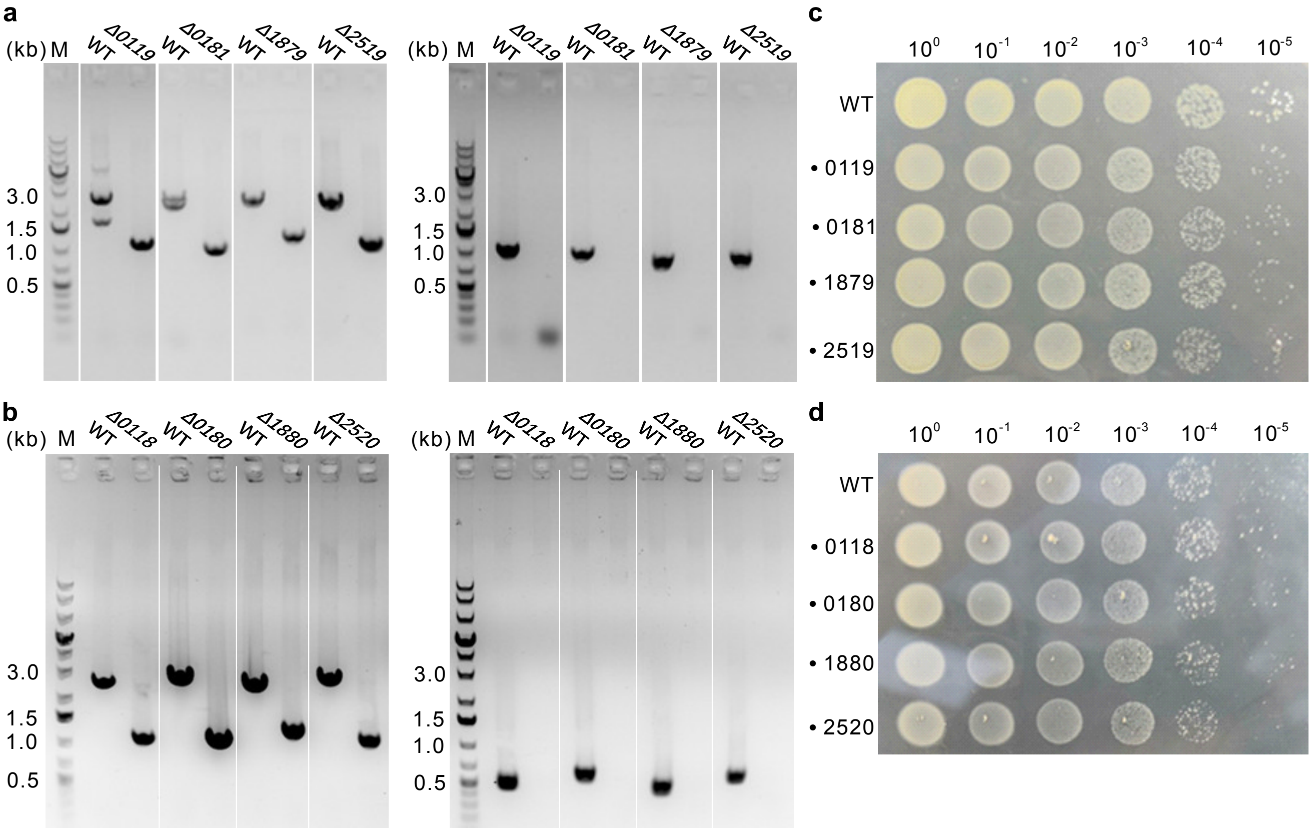

Supplementary Figure 9. Construction of the ATPase and TadC knockout strains

PCR verification of the ATPase (**a**) and TadC (**b**) knockout strains using the Flanking (Left) and gene specific (right) primers. Spot test to show the growth of the ATPase (**c**) and TadC (**d**) knockout strains. Knockout of the ATPase or TadC genes alone exhibited no obvious growth difference comparing with the WT strain on solid medium

Supplemental Table 1. Plasmids used in this study

Plasmid	Description	Reference
pGE	*Genome-editing plasmid*	Li et al[1]
pGE-*sire_0119*	*sire_0119* (ATPase gene) knockout	This study
pGE-*sire_0181*	*sire_0181* (ATPase gene) knockout	This study
pGE-*sire_1879*	*sire_0181* (ATPase gene) knockout	This study
pGE-*sire_2519*	*sire_2519* (ATPase gene) knockout	This study
pGE-*sire_0118*	*sire_0118* (TadC gene) knockout	This study
pGE-*sire_0180*	*sire_0180* (TadC gene) knockout	This study
pGE-*sire_1880*	*sire_1880* (TadC gene) knockout	This study
pGE-*sire_2520*	*sire_2520* (TadC gene) knockout	This study

Supplemental Table 2. Strains used in this study

**Strain**	**Phenotype**	**Reference**
S. islandicus REY15A	*Wild type*	Guo et al[2]
Sis E2333	REY15A *ΔpyrEFΔlacS*	Deng et al[3]
Sis/pGE	Control for knockout	Liu et al[4]
Δsire_0119	*sire_0119* knockout	This study
Δsire_0181	*sire_0181* knockout	This study
Δsire_1879	*sire_0181* knockout	This study
Δsire_2519	*sire_2519* knockout	This study
Δsire_0118	*sire_0118* knockout	This study
Δsire_0180	*sire_0180* knockout	This study
Δsire_1880	*sire_1880* knockout	This study
Δsire_2520	*sire_2520* knockout	This study

Supplemental Table 3. Spacers selected and used in this study

Name	Sequence (5’-3’)	Source
0119-S-F	AAGCATACGAACAAGTTCATACCCTCGTCTAACATCATCCATA	This study
0119-S-R	AGCTATGGATGATGTTAGACGAGGGTATGAACTTGTTCGTATG	This study
0181-S-F	AAGAATGGCGATAGGATTGCCGCAACATTCAGACGTGAAGTAT	This study
0181-S-R	AGCATACTTCACGTCTGAATGTTGCGGCAATCCTATCGCCATT	This study
1879-S-F	AAGATAAGTCCTATATTATTGATTAAAAACGTATCGATAAGTT	This study
1879-S-R	AGCAACTTATCGATACGTTTTTAATCAATAATATAGGACTTAT	This study
2519-S-F	AAGGAAAAGCCTCTAACGATTATTGACTTGGTTTACAAGTATG	This study
2519-S-R	AGCCATACTTGTAAACCAAGTCAATAATCGTTAGAGGCTTTTC	This study
0118-S-F	AAGTCAGAGACTCTTTTAAGTTGCTTACTATCGCATAATTTCT	This study
0118-S-R	AGCAGAAATTATGCGATAGTAAGCAACTTAAAAGAGTCTCTGA	This study
0180-S-F	AAGTCTCTCTTTGGTTCAGTCCCATTTCCTATAATGGCTATTT	This study
0180-S-R	AGCAAATAGCCATTATAGGAAATGGGACTGAACCAAAGAGAGA	This study
1880-S-F	AAGATGATTTTACTGGCCTTTCAGTATATTTCGTCAACAATAA	This study
1880-S-R	AGCTTATTGTTGACGAAATATACTGAAAGGCCAGTAAAATCAT	This study
2520-S-F	AAGGTTTCGTTGGAGGTTTTACTCAGAAAGAACCTCATTCCTA	This study
2520-S-R	AGCTAGGAATGAGGTTCTTTCTGAGTAAAACCTCCAACGAAAC	This study

Supplemental Table 4. Oligonucleotides used in this study

Name	Sequence (5'-3')	Source
**Oligonucleotides used to amplify the L- and R-arm of the corresponding genes of interest**		
0119-L-arm-F-Sph I	AAGTACAATTGTGCT**GCATGC**CTATTAACCTAGCAGAATAA	This study
0119-L-arm-R	TCCCTATCGAGGGCTTAACCTTCTTTATGACGAATTAAAC	This study
0119-R-arm-F	GTTTAATTCGTCATAAAGAAGGTTAAGCCCTCGATAGGGA	This study
0119-R-arm-R-Xho I	TAACATATTGGATG**CTCGAG**AAAAAGATGAAAGATGTAAA	This study
0181-L-arm-F-Sph I	AAGTACAATTGTGCT**GCATGC**AGTAGAGTTTCCTTAATTTC	This study
0181-L-arm-R	ATTGTTAATCTTGGTTTCAATATATATCACTTCTCCATCT	This study
0181-R-arm-F	AGATGGAGAAGTGATATATATTGAAACCAAGATTAACAAT	This study
0181-R-arm-R-Xho I	TAACATATTGGATG**CTCGAG**TACCCTAACTGATTTATATA	This study
1879-L-arm-F-Sph I	AAGTACAATTGTGCT**GCATGC**GAGATGGGTATGATTTGGTC	This study
1879-L-arm-R	ATTTCTGAAATTGGTATTTCTTCGCTTACTTTATAATAGC	This study
1879-R-arm-F	GCTATTATAAAGTAAGCGAAGAAATACCAATTTCAGAAAT	This study
1879-R-arm-R-Xho I	TAACATATTGGATG**CTCGAG**TAGAATTCCTTATTTCCTCG	This study
2519-L-arm-F-Sph I	AAGTACAATTGTGCT**GCATGC**GTTGCAATAGCTGCGGTCAG	This study
2519-L-arm-R	TCCTTACTCCAGCCTAATAACTCTAAGAGATATTATTCCC	This study
2519-R-arm-F	GGGAATAATATCTCTTAGAGTTATTAGGCTGGAGTAAGGA	This study
2519-R-arm-R-Xho I	TAACATATTGGATG**CTCGAG**CTATGAAACCTAATCTACTG	This study
0118-L-arm-F-Sph I	AAGTACAATTGTGCT**GCATGC**GATGGTCTCAATTTTAGAGA	This study
0118-L-arm-R	GATATGCCAAGAATATTTAGATATATTTACCTAAAATTAC	This study
0118-R-arm-F	GTAATTTTAGGTAAATATATCTAAATATTCTTGGCATATC	This study
0118-R-arm-R-Xho I	TAACATATTGGATG**CTCGAG**TGTAGCATTCCAAGCAATGC	This study
0180-L-arm-F-Sph I	AAGTACAATTGTGCT**GCATGC**AAAAACCGTAAGGGATTATA	This study
0180-L-arm-R	AGAGTGCCGAAATAGAGATTCCTATTGTTACTTTCTCAGA	This study
0180-R-arm-F	TCTGAGAAAGTAACAATAGGAATCTCTATTTCGGCACTCT	This study
0180-R-arm-R-Xho I	TAACATATTGGATG**CTCGAG**TCATTTACTGGTGGTTCCAA	This study
1880-L-arm-F-Sph I	AAGTACAATTGTGCT**GCATGC**GACTATCATTAAGATATAGG	This study
1880-L-arm-R	ATAATCAAATCTAAGTTACCTCAAATAGCCTTTGTCTCAA	This study
1880-R-arm-F	TTGAGACAAAGGCTATTTGAGGTAACTTAGATTTGATTAT	This study
1880-R-arm-R-Xho I	TAACATATTGGATG**CTCGAG**TACAATATTCTTAGTCCCAT	This study
2520-L-arm-F-Sph I	AAGTACAATTGTGCT**GCATGC**GGGAAGTAAGAGGGAAAGAA	This study
2520-L-arm-R	ATATGAACATTTCAGCTAGGCTTCTTTCAGTCACCCCTAT	This study
2520-R-arm-F	ATAGGGGTGACTGAAAGAAGCCTAGCTGAAATGTTCATAT	This study
2520-R-arm-R-Xho I	TAACATATTGGATG**CTCGAG**TCTGTCCGTTAAATGTGGTT	This study
**Oligonucleotides used to verify the knockout strains**		

0119-Flanking-F	GGTAAGGGAGCTATTTAATGTAGC	This study
0119-Flanking-R	CTACAGAACAAACTAGCAAAGACTA	This study
0119-int-F	CTGCAGGATCATATGTAAATGCTGG	This study
0119-int-R	CTAAGCAAGATAAGAATATCCGTACC	This study
0181-Flanking-F	GATAGCCTCAGAGAGAATATTGGA	This study
0181-Flanking-R	GATATTGTAGTTTATACCTTAGCCC	This study
0181-int-F	GCCGAATCCTCACATCTTTATTACC	This study
0181-int-R	CTCTTGTCCTCTTATCTCACCTAC	This study
1879-Flanking-F	ACGAGATTTTCGAATGGAAGGAGTTA	This study
1879-Flanking-R	GTAACATAGCCTCTCTTCTTAAACC	This study
1879-int-F	GAGCTAACTGTTCCCTTAGCAGAT	This study
1879-int-R	CACTGGCTTGAATCTTAGTTTACC	This study
2519-Flanking-F	GGCTTAGAAAACGCTTTAGTTACTG	This study
2519-Flanking-R	CTCTTAGTACGCTCGCTTATCCA	This study
2519-int-F	CGAACCACAGCTTGATCAGAGAGA	This study
2519-int-R	CATGACCACTGGCAACAGCTTGA	This study
0118-flanking-F	GTCATGGCCCAGTTTACACTA	This study
0118-flanking-R	GGAGAAATTAGAGATAGAGAAGG	This study
0118-int-F	GTACTTTGCTTAACATGCTCAC	This study
0118-int-R	GGAACTACTCTAATAACACAGC	This study
0180-flanking-F	GGAATCTCTGGAATTAGGATAG	This study
0180-flanking-R	GAAGAGGTAGAGAGAGAATTAC	This study
0180-int-F	CACAGCAATGCGCTACGCTAG	This study
0180-int-R	GTATTTGCTGTACACCACCATTC	This study
1880-flanking-F	GCTAGACAATCAATCTCTTCCC	This study
1880-flanking-R	CACGTTCTGAACAACACTATACG	This study
1880-int-F	GTGGCGAGTCAATAGGAATTTC	This study
1880-int-R	CATCAGCGACCGATATCTTAC	This study
2520-flanking-F	CAGACTCTCCTTAAGATATAGAC	This study
2520-flanking-R	GGTCCAATTACTATTGCATCTCC	This study
2520-int-F	GTAAGAGACAGCATCCAAGATG	This study
2520-int-R	CATATATCTAGGTCTATTGCTAGC	This study
**Oligonucleotides used to determine the transcriptional level of the corresponding genes by RT-qPCR**		
0119-qPCR-F	GCTACATTACCTTCTCTATCTC	This study
0119-qPCR-R	CAGAAGTGACTAGAGAAACAG	This study

0181-qPCR-F	GTTCTTCAGTAGTGATACGAAG	This study
0181-qPCR-R	GTAACTCCTATTGACATGATACT	This study
1879-qPCR-F	GTCATAGGTTCTACCGGATC	This study
1879-qPCR-R	CCTTATCCAGTTATCATGAACTA	This study
2519-qPCR-F	GAAAGACCACTGCATTAAACTC	This study
2519-qPCR-R	CATAGTTTGAAGGTCTAGTATAG	This study
0118-qPCR-F	CTATTAGGAGTTGGTCTTAGG	This study
0118-qPCR-R	CTTAGTACTGCCATCAGAGAC	This study
0180-qPCR-F	GAAACTTTAGCTTCTACTGCTG	This study
0180-qPCR-R	GCCATTATAGGAAATGGGACTG	This study
1880-qPCR-F	GTCAATAGGAATTTCCATGCC	This study
1880-qPCR-R	CCAGTAAAATCATTGGAGTGAC	This study
2520-qPCR-F	CTCCACTTGACGCTTTAGAG	This study
2520-qPCR-R	CAACGAAACTGGAGCCAATTAAG	This study
TBP-F	GTGGCAACAGTTACGTTAGAG	Liu et al[5]
TBP-R	CCTTGGGCTGTTCTAATCTG	Liu et al[5]

Supplementary References

1. Li Y*, et al.* Harnessing Type I and Type III CRISPR-Cas systems for genome editing. *Nucleic Acids Res* **44**, e34 (2016).

2. Guo L*, et al.* Genome analyses of Icelandic strains of Sulfolobus islandicus, model organisms for genetic and virus-host interaction studies. *J Bacteriol* **193**, 1672-1680 (2011).

3. Deng L, Zhu H, Chen Z, Liang YX, She Q. Unmarked gene deletion and host-vector system for the hyperthermophilic crenarchaeon Sulfolobus islandicus. *Extremophiles* **13**, 735-746 (2009).

4. Liu J*, et al.* Archaeal extracellular vesicles are produced in an ESCRT-dependent manner and promote gene transfer and nutrient cycling in extreme environments. *ISME J* **15**, 2892-2905 (2021).

5. Liu J*, et al.* Virus-induced cell gigantism and asymmetric cell division in archaea. *Proc Natl Acad Sci U S A* **118**, (2021).
